# Diabetic Microvascular Complications: Novel Risk Factors, Biomarkers, and Risk Prediction Models

**DOI:** 10.1155/2016/2172106

**Published:** 2015-12-08

**Authors:** Gareth J. McKay, Boon Wee Teo, Ying-Feng Zheng, Usha Sambamoorthi, Charumathi Sabanayagam

**Affiliations:** ^1^Centre for Public Health, Queen's University Belfast, Belfast BT12 6BA, UK; ^2^Department of Medicine, Yong Loo Lin School of Medicine, National University of Singapore, Singapore 119228; ^3^Health Outcomes Research, Department of Pharmaceutical Systems and Policy, West Virginia University, Morgantown, WV 26505, USA; ^4^State Key Laboratory of Ophthalmology, Zhongshan Ophthalmic Center, Sun Yat-sen University, Guangzhou 510275, China; ^5^Singapore Eye Research Institute, Singapore 169856; ^6^Office of Clinical Sciences, Duke-NUS Graduate Medical School, Singapore 168751

The prevalence of diabetes mellitus is increasing worldwide paralleling the rise in obesity, sedentary lifestyle, and increased life expectancy. It is estimated that globally 382 million people live with diabetes and the International Diabetes Federation expects this number to rise to 592 million by 2035. Persons with diabetes are at increased risk of developing microvascular complications such as diabetic retinopathy (DR), a leading cause of blindness, diabetic nephropathy (DN), a leading cause of renal failure, and diabetic peripheral neuropathy (DPN), a leading cause of diabetic foot disorders and lower limb amputations, and all are major causes of morbidity and deaths. As many older persons with diabetes are living longer, the prevalence of these microvascular complications is also on the rise despite improvements in control of glycemia, blood pressure, and lipid levels. Given that these microvascular complications are largely preventable, it is important to identify novel risk factors or biomarkers associated with early detection of onset/progression of DR, DN, and DPN to mitigate against these microvascular complications.

Several biomarkers associated with the pathogenic mechanisms that underlie these microvascular complications including inflammation, endothelial dysfunction, oxidative stress, hemostasis, microangiopathy, proteomic and metabolomic markers, and those representing end-organ damage have facilitated improved risk stratification besides providing insight into the underlying etiology of the disease. This special issue contains six papers which examine some of the critical issues in the identification and understanding of novel risk factors and their potential in the identification and risk stratification of diabetic microvascular complications.

The paper by Q. Ma and colleagues entitled “Regular Chinese Green Tea Consumption Is Protective for Diabetic Retinopathy: A Clinic-Based Case-Control Study” examines the association between regular Chinese green tea consumption and the risk of DR in persons with diabetes from China. Green tea is a popular beverage in East Asia and contains huge amount of polyphenols with strong antioxidant activity and anti-inflammatory properties with evidence in support of clinical benefit across a range of chronic conditions. The authors observed that regular consumption of Chinese green tea for at least a year reduced the risk of DR by up to 50% compared to those who did not consume green tea. The study findings if confirmed in prospective studies with larger sample size suggest regular Chinese green tea consumption may offer a cheap and easy-to-operate approach for preventing DR in low-income countries.

J. Jin and colleagues present a paper in this special issue entitled “Development of Diagnostic Biomarkers for Detecting Diabetic Retinopathy at Early Stages Using Quantitative Proteomics.” They performed a comprehensive proteomic analysis of DR to identify novel biomarker candidates that are specifically expressed within the human vitreous. This was achieved through data-mining of previously published DR-related studies as well as* de novo* analysis of novel experimental data. The* in silico* analysis identified 96 plausible candidates from previously published literature which following* de novo* verification in plasma samples from persons with diabetes identified 11 proteins showing significant differences and a final 4-protein multimarker panel including ITIH2, APOA4, C7, and CLU significantly improved the risk prediction models between no DR and both proliferative and nonproliferative DR. The proposed systematic bioinformatics and* de novo* proteomic analysis pipeline has identified novel biomarkers associated with DR with strong discriminatory power offering future potential for clinical utility.

The paper by C. W. Wong and colleagues entitled “Serum Cystatin C, Markers of Chronic Kidney Disease, and Retinopathy in Persons with Diabetes” examines the potential for cystatin C to predict DR outcomes better than renal function measures derived from serum creatinine and albuminuria. Chronic kidney disease (CKD) defined by the three markers of serum creatinine, serum cystatin C, and albuminuria has been shown to improve prediction of renal failure and mortality. The aim of this study was to determine if CKD defined by all three markers (creatinine, albuminuria, and cystatin C) was more strongly indicative of moderate DR than each marker in isolation or in dual combination. The study concluded that CKD defined by a triple marker panel was strongly associated with moderate DR in Indian adults with diabetes. If confirmed by future prospective studies and in other populations, a triple marker approach may have implications on changing clinical practice to incorporate cystatin C to improve risk stratification of DR in persons with CKD. This group of high-risk individuals may benefit from closer surveillance and more timely intervention before the onset of irreversible sight threatening complications.

E. M. Lipner and colleagues present a paper entitled “Linkage Analysis of Genomic Regions Contributing to the Expression of Type 1 Diabetes Microvascular Complications and Interaction with HLA.” Evidence for familial inheritance in diabetic complications has been clearly demonstrated, suggesting a genetic contribution to these phenotypes. Although many linkage and association studies have focused on identifying T1D susceptibility loci, elucidation of the underlying genetic architecture of diabetic complications has proved elusive. Few genetic linkage studies have focused beyond renal complications of T1D and the study by E. M. Lipner and colleagues is the first to use linkage analysis to investigate the influence of the HLA region on the manifestation of diabetic complications. Their study has used a large well-characterized cohort of T1D families to identify gene-loci that predispose to T1D complications focusing specifically on a previously identified region of chromosome 6. The “any complication” phenotype identified three loci, including the HLA locus and two novel complications-related loci on chromosome 6, providing strong support for inherited influences that contribute to the manifestation of diabetic complications.

G. J. McKay and colleagues present a paper entitled “Bioinformatic Evaluation of Transcriptional Regulation of WNT Pathway Genes with Reference to Diabetic Nephropathy.” Members of the WNT/*β*-catenin pathways have been previously implicated in the characteristic DN disease processes of interstitial fibrosis and glomerular sclerosis. These processes are partially controlled by transcription factors (TFs), proteins that bind to gene promoter regions attenuating their regulation. This paper sought to identify whether predicted cis-acting transcription factor binding sites (TFBS) are overrepresented within the promoter regions of WNT pathway members compared to genes across the genome. Three TFBS profiles were found to be significantly enriched within the WNT pathway genes examined and their expression profiles significantly altered in publically available DN-related datasets. This study highlights the effectiveness of an* in silico* pipeline to identify key regulators of this pathway. Modification of TF binding to gene promoter regions implicated in DN pathology may reduce disease progression, making refinement of targeted therapeutic strategies possible through clearer delineation of their role.

The paper by J. McEneny and colleagues entitled “A Cross-Sectional Study Demonstrating Increased Serum Amyloid A Related Inflammation in High-Density Lipoproteins from Subjects with Type 1 Diabetes Mellitus and How This Association Was Augmented by Poor Glycaemic Control” examines whether HDL-related inflammation is increased in subjects with type 1 diabetes mellitus (T1DM). Inflammatory atherosclerosis is increased in subjects with T1DM. Normally high-density lipoproteins (HDL) protect against atherosclerosis; however, in the presence of serum amyloid A (SAA) related inflammation, this property may be reduced. SAA augments atherogenesis by enhancing the binding of HDL to the arterial wall. Authors observed increased SAA related inflammation in subjects with T1DM that was augmented by poor glycemic control. The study findings suggest that SAA is a useful inflammatory-biomarker in T1DM, which may contribute to the accelerated atherosclerosis risk in these subjects.

In summary, the complexity of the task required to better understand the pathogenic mechanisms associated with diabetic complications looms large with many important questions still remaining unanswered. We hope that the papers presented in this special issue will help inform the research agenda necessary for future progress in this field.

*Gareth J. McKay* *Gareth J. McKay* *Boon Wee Teo* *Boon Wee Teo* *Ying-Feng Zheng* *Ying-Feng Zheng* *Usha Sambamoorthi* *Usha Sambamoorthi* *Charumathi Sabanayagam* *Charumathi Sabanayagam*

